# Prevalence of and factors associated with dental anxiety among medical and dental students of the Northern State Medical University, Arkhangelsk, North-West Russia

**DOI:** 10.1080/22423982.2018.1454786

**Published:** 2018-03-22

**Authors:** Sergei N. Drachev, Tormod Brenn, Tordis A. Trovik

**Affiliations:** a Department of Community Medicine, Faculty of Health Sciences, UiT The Arctic University of Norway, Tromsø, Norway; b International School of Public Health, Northern State Medical University, Arkhangelsk, Russia

**Keywords:** Dental anxiety, medical and dental students, North-West Russia

## Abstract

The objective was to assess the prevalence of and factors associated with dental anxiety (DA) in medical and dental students in North-West Russia. This cross-sectional study included 422 medical and 285 dental undergraduate Russian students aged 18–25 years from the Northern State Medical University in Arkhangelsk. Corah’s Dental Anxiety Scale (DAS) was applied to measure DA. Information on socio-demographic and socioeconomic factors, oral health behaviour and general and oral health was obtained from a structured, self-administered questionnaire. A clinical examination was performed to assess caries experience, Simplified Oral Hygiene Index, and Gingival Index. DAS score ≥13 was found in 13.7% and 2.2% of medical and dental students, respectively. Female sex (incidence rate ratio [IRR] = 1.11, *p* = 0.013), lower education of mother (IRR = 1.13, *p* = 0.001), and poor self-assessed oral health (IRR = 1.15, *p* < 0.001) were associated with DA in medical students. Corresponding factors in dental students were female sex (IRR = 1.16, *p* = 0.001), irregular dental visits (IRR = 1.19, *p* = 0.001), infrequent tooth-brushing (IRR = 1.17, *p* = 0.007), pain in mouth (IRR = 1.09, *p* = 0.031) and number of missing teeth (IRR = 1.13, *p* = 0.007). The prevalence of high DA was lower in dental students than in medical students. DA was associated with sex, mother’s education, poor oral health behaviour and self-assessed and clinically assessed oral health.

## Introduction

Oral health is an integral part of general well-being and a significant public health issue. Despite increased awareness among dentists and patients of preventive approach to oral diseases, and innovations in dental equipment and pain reduction, dental anxiety (DA) remains an important problem in clinical dentistry [1]. DA is described as a state of excessive and unreasonable apprehension that “something dreadful is going to happen in relation to dental treatment, and it is coupled with a sense of losing control” [2]. Dental fear is related to DA and is described as a normal unpleasant emotional reaction to perceived threat or danger in a dental situation [1]. The concepts of dental fear and DA are frequently used interchangeably in dental studies, implying “strong negative feelings associated with dental treatment” [1,2]. Several psychometric tests have been developed to differentiate people with and without DA. Along with single-item questions, Corah’s Dental Anxiety Scale (DAS) [3], the Modified Dental Anxiety Scale (MDAS) [4], and Kleinknecht’s Dental Fear Survey [5] are the most commonly used tools in epidemiological studies to measure DA in adults [6,7], although none of the existing instruments are regarded as a gold standard [6]. The prevalence of high DA varies from 2% to 30% worldwide depending on the study population, the methods applied, and the cut-off scores used [1,8]. There is strong evidence that DA is associated with dental attendance; it has been reported that individuals with higher DA tend to visit the dentist irregularly [9,10], which in turn may lead to a deterioration in oral health. Studies have demonstrated that DA is associated with poor self-reported [8,9] and clinically assigned [11,12] oral health, more decayed and missing teeth [10,11], fewer filled teeth [10,12] and worse periodontal health [13]. In addition, DA has been related to poor self-reported general health [8], psychological disorders [14], particular temperamental or psychological traits [7] and lower education [8,12].

Several reports showed that younger individuals are more likely to experience DA than middle-aged and elderly adults [10,12]. Many studies have focused on DA in young university students [15–26]. Lower DA has been found in dental than in non-dental students [15–17], and further reductions were shown among dental students during their dental training [17,18]. Reported predictors for DA have included self-reported need for dental treatment, tobacco use, abnormal attitudes towards food, insufficient oral hygiene, less frequent dental visits and the anticipation of pain [19,24,25]. No relationships between DA and clinically assigned oral health have been studied in young university students, but some studies on other factors showed that female students had higher DA than male students [16,19–22], whereas other studies found no sex differences [15,23,24].

Epidemiological studies have shown considerably poorer oral health among populations living in Russian circumpolar areas than in other Russian areas [27]. Nevertheless, we found only one study on DA in Russia, which was conducted in St. Petersburg in 1992, more than 20 years ago [28]. The study included 288 urban schoolchildren aged 13 to 18 years and yielded a 12.6% prevalence of high DA. Sex, treatment and toothache experience, dental fear in the family and fear at first dental visit were associated with high DA. At present, there is no information available on the prevalence of DA and the association between DA and oral health behaviour, general health and oral health status in young adults living in the northern parts of Russia.

The aims of this study are to assess the prevalence of DA and to explore the association between DA and socio-demographic and socioeconomic factors, oral health behaviour and general and oral health in medical and dental students attending the Northern State Medical University (NSMU) in Arkhangelsk, North-West Russia.

## Material and methods

### Study setting and population

During the 2015–2016 academic year, approximately 3900 students, mainly from the European North-West of Russia (the regions of Arkhangelsk, Vologda and Murmansk; the Komi and Karelia Republics; and the Nenets Autonomous Okrug), attended the NSMU. In this cross-sectional study, we invited full-time undergraduate students from two faculties: 1) medical (*n* = 1482), which included students from the departments of general medicine and paediatric medicine; and 2) dental (*n* = 524). Combined, these faculties make up ~51.4% of the total number of students at the NSMU. For convenience, students from other non-medical faculties and smaller medical faculties and departments (medical biochemistry, medical prophylaxis, pharmacy) were not considered. Students from the international faculty of general practitioners were also not invited, as we focused on students of Russian nationality only.

### Sampling

We applied a two-stage sampling technique for enrolment. In Stage 1 (recruitment + questionnaire 1 [Q1]), medical and dental students from each year of education (6 years for medical students; 5 years for dental students) were informed about the study and invited to participate at the end of a randomly selected scheduled classroom lecture. Altogether, 1579 students (1142 medical and 437 dental students) attended the lectures, of whom 1385 (965 medical and 420 dental students) agreed to participate, signed the informed consent and completed the self-administered, anonymous Q1 in Russian.

In Stage 2 (questionnaire 2 [Q2] + dental examination), all dental students (*n* = 420) and a stratified, random sample of medical students (*n* = 823) were invited by phone, using the contact mobile numbers collected in Stage 1. If a student did not answer at the first call, one additional call was placed on a different day. Students who agreed to participate completed a second, self-administered, anonymous Q2 and underwent a dental examination (total *n* = 807). The exclusion criteria were: age under 18 or over 25 years, non-Russian nationality, presence of fixed orthodontics bands and pregnancy. The response rate was 57.6% (range: 41.5–69.1% within different years of education) and 79.3% (range: 70.3–85.4%) in medical and dental students, respectively. Only students with no missing data (*n* = 707) were included in statistical analysis. The sampling has been described previously in detail [29].

### Instruments

Q1 gathered information on socio-demographic factors, socioeconomic factors and oral health behaviours. Age group (18–20/21–25 years), sex, faculty (medical/dental) and place of childhood residence (urban/rural) were considered as socio-demographic variables. Whether the students were eligible for free education (yes/no), which is generally representative of students with higher grades on their entrance exams, was used as a socioeconomic variable. A university applicant who has failed in competition to be admitted at the NSMU can still study there, but they have to pay tuition each year. Students reporting dental visits at least once every 6 months or once a year were categorised as having regular dental visits, and those who said they visited the dentist occasionally or had no visits in the last 3 years were categorised as having irregular dental visits. Frequency of tooth-brushing was categorised as less than twice a day (consisting of the responses: never, less than once a week, once every few days and once a day) and twice a day or more. The variable “skipping tooth-brushing” was categorised as *no* when students reported skipping tooth-brushing never or almost never, and as *yes* when skipping tooth-brushing was reported sometimes during a week, every day or almost every day. The variable “toothpaste” was split into two categories: with fluoride and without fluoride/difficult to answer. The students were also asked about their oral health. Students who rated their oral health as excellent, very good or good were categorised as having good self-reported oral health, and those who rated their oral health as fair or poor were categorised as having poor self-reported oral health. The variables “experienced pain in mouth” and “experienced gum bleeding during tooth-brushing” were also dichotomised into *no* when students responded never or rarely, and *yes* when students responded sometimes, often or always.

In Q2, students were asked three global questions about their health: “Overall, how would you rate your general health/your psychological health/your ability to cope with different aspects of life?” Responses were given on a 5-point scale: (1) excellent, (2) very good, (3) good, (4) fair and (5) poor. For analysis, each variable was dichotomised as “good” (1–3) and “poor” (4,5). Information was also gathered about mother’s education and subjective socioeconomic status (SES). Mother’s education was split into lower than university (high school: 9–11 years of school; specialised secondary: professional medical or pedagogical college, technicum) and university. The respondents rated the SES of their family in accordance with socioeconomic indicators (education, occupation, income) using the 10-step MacArthur Scale of Subjective Social Status, for which 10 indicates “best off” and 1 indicates “worst off” [30]. The median SES (6.0) was used as the cut-off to dichotomise this variable into “low SES” (1–5) and “high SES” (6–10). The questions on regularity of dental visits; self-reported oral and general health characteristics; and mother’s education included the response option “difficult to answer”. When that response was chosen in either questionnaire (Q1: *n* = 48, Q2: *n* = 11), this data was considered missing and the participants were excluded from the analysis.

### Validity and reliability of dental anxiety scale inventory

In Q2, the four-item Corah’s DAS was used to assess DA [3]. The English version of DAS was translated/back-translated into Russian/English by two bilingual individuals independently, and the conceptual and functional equivalence of the instrument was verified by colleagues at the NSMU. Before the study began, the questionnaire was pilot-tested on 12 students aged 18 to 25 years who did not participate in the study, after which only minor changes were required. Students answered each item on a 5-level scale, and the total DAS score was calculated as the sum of the four items and ranged from 4 to 20. A DAS score of 13 or more was considered a high DA [31].

That fact that only three of the 807 respondents who answered the DAS questions omitted one item adds support to the face validity. Students who confirmed DA as their reason for not getting a dentist appointment had significantly higher DAS scores, compared to students who reported “other” reasons for not going to a dentist (12.5 vs. 8.5, *p* < 0.001), which provided evidence of criterion validity. Good reliability of the DAS in terms of the inter-item correlation coefficient (Cronbach’s alpha = 0.85) was determined. If a single item was removed, the Cronbach’s alpha value decreased compared to its original undeleted value. The average of the inter-item correlation among the DAS items was 0.59 (range: 0.47–0.72), with no negative correlations. The corrected item-total correlations ranged from 0.63 to 0.78, and all values were above the minimum recommended level of 0.20 for including an item into a scale [32].

### Clinical dental examination

A clinical dental examination without radiographs was performed at the Dental Clinic of the NSMU from February to May 2016. One researcher (SND) executed all clinical examinations in accordance with World Health Organization (WHO) recommendations [33], and an assistant filled in the details on the clinical sheet. All permanent teeth, excluding third molars, were taken into account during the clinical examination. Dental caries experience was measured by the DMFT index, which is the sum of decayed teeth (DT), missing teeth due to caries (MT) and filled teeth (FT). The Simplified Oral Hygiene Index (OHI-S) proposed by Green & Vermillion (1964) was used to assess oral hygiene [34]. The total score of this index was calculated as the sum of the average individual debris and calculus scores. For the assessment of the qualitative changes in the gingival soft tissue, we employed the Gingival Index (GI) of Loe & Silness [35]. Six index teeth (44/32/36/24/12/16) and four areas for each tooth (mesial, distal, buccal and lingual) were considered to calculate GI.

Before the study start, the researcher was calibrated at the Dental Clinic of UiT The Arctic University of Norway, Tromsø, Norway, according to WHO standards [33]. In June 2016, 54 students were selected randomly for clinical re-examination. Intraclass correlation coefficients for DMFT and GI were 0.989 (95% confidence interval [CI]: 0.981–0.993) and 0.828 (95% CI: 0.721–0.896), respectively.

### Statistical analysis

Given the skewed distribution of the DAS score, the Mann-Whitney U test was used for two independent groups of studied variables. Simple Poisson regression was carried out to assess crude associations between DAS scores (dependent count variable) and scores from clinical dental examinations. Given the non-significant test for alpha, negative binomial regression did not fit our data better than Poisson regression.

Multivariable Poisson regression with robust estimates was used, with the DAS score as the dependent variable. Only independent variables with *p*-values less than 0.2 in univariable analysis were included in the multivariable model. Backward stepwise selection was used to find significant independent variables associated with the DAS score. Significance levels for removal and addition to the final model were chosen as 0.2 and 0.1, respectively. Given the significant interactions between “faculty” and “mother’s education” and “faculty” and “regularity of dental visits”, analyses were performed for medical and dental students separately.

Descriptive statistics and univariable analyses were performed with IBM SPSS Statistics for Macintosh version 23.0 (Armonk, NY: IBM Corp.). Poisson regression was done with STATA version 14.0 (StataCorp, College Station, Texas, USA). The level of significance for testing all statistical hypotheses was set at *p* = 0.05.

### Ethical considerations

The study was approved by the Regional Ethical Committee of Norway (2015/1788/REK nord) and the Ethical Committee of the NSMU, Russia (№ 05/10–15 from 19.10.2015).

## Results

There were no significant differences in age nor sex between students participating in Stage 1 (*n* = 1385) and Stage 2 (*n* = 807). Likewise, the 707 students included in the analysis did not differ by age, sex, or subjective SES from students who were excluded from the analysis due to missing data (*n* = 100). Mean age was 20.2 years (standard deviation [SD] 1.6).

**Figure 1.**
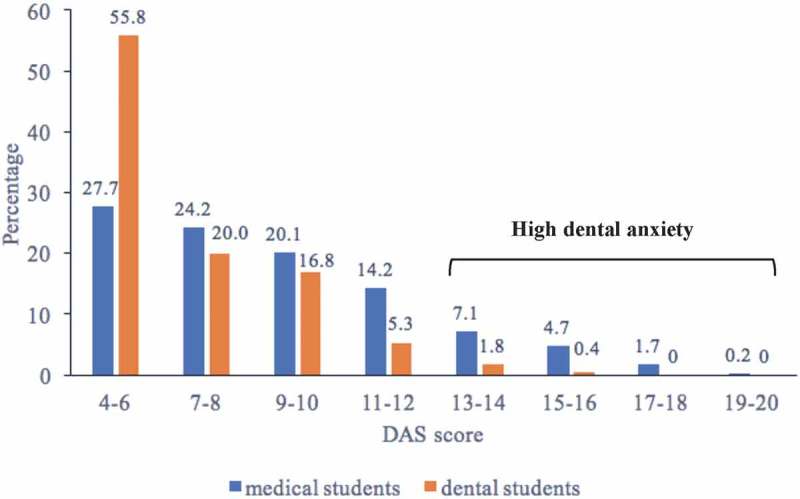
Distribution of the Dental Anxiety Scale (DAS) score in medical students (*n* = 422) and dental students (*n* = 285).

Medical students had a higher mean DAS score than dental students (8.81, SD 3.23 vs. 6.73, SD 2.36; *p* < 0.001). The prevalence of high DA (DAS ≥13) was 13.7% and 2.2% in medical and dental students, respectively (*p* < 0.001) (Figure 1). Compared to dental students, medical students were older (44.8% vs. 35.4% in the age group of 21–25 years; *p* = 0.013), were more often eligible for free education (87.9% vs. 67.7%; *p* < 0.001), and reported a university mother’s education less often (50.2% vs. 58.9%; *p* = 0.023). In medical students, women had a higher mean DAS score than men, whereas students from urban areas, those with higher subjective SES, and those whose mothers had a university education had a lower mean DAS score. There were no differences in DAS score among medical students in different age groups or among those who were and were not eligible for free education. In dental students, no statistically significant differences in DAS score were observed across all socio-demographic and socioeconomic characteristics considered (Table 1).

**Table 1.** Socio-demographic and socioeconomic characteristics associated with dental anxiety among medical and dental students in Arkhangelsk, Russia.

	Medical students (*n* = 422)			Dental students (*n* = 285)		
	*n*	DAS score (SD)	p*	*n*	DAS score (SD)	p*
**Age group (years)**			0.470			0.382
18–20	233	8.67 (3.13)		184	6.85 (2.50)	
21–25	189	8.97 (3.34)		101	6.50 (2.08)	
**Sex**			0.005			0.091
Male	97	8.04 (3.01)		81	6.31 (2.09)	
Female	325	9.04 (3.26)		204	6.89 (2.44)	
**Place of childhood residence**			0.019			0.729
Urban	304	8.62 (3.29)		202	6.72 (2.30)	
Rural	118	9.29 (3.01)		83	6.73 (2.52)	
**Eligible for free education**			0.764			0.114
Yes	371	8.82 (3.22)		193	6.84 (2.31)	
No	51	8.71 (3.30)		92	6.48 (2.46)	
**Subjective SES**			0.030			0.868
Less than 6.0	146	9.21 (3.17)		93	6.73 (2.46)	
6.0 and more	276	8.59 (3.24)		192	6.72 (2.32)	
**Mother’s education**			<0.001			0.854
<University	210	9.39 (3.25)		117	6.74 (2.40)	
University	212	8.23 (3.10)		168	6.71 (2.34)	

DAS: Dental Anxiety Scale; SD: Standard Deviation; SES: socioeconomic status. *p from the Mann-Whitney U test.

When looking at oral health behaviour, differences were found between medical and dental students who reported regular dental visits (77.5% vs. 84.9%; *p* < 0.001), brushed their teeth twice a day or more (75.4% vs. 86.7%; *p* < 0.001), skipped tooth-brushing (37.9% vs. 28.1%; *p* = 0.007) and used a toothpaste with fluoride (40.3% vs. 56.5%; *p* < 0.001). Both medical and dental students who reported regular dental visits had a lower DAS score compared to those who reported irregular dental visits. No differences in DAS score were found between categories of tooth-brushing, skipping tooth-brushing and using toothpaste with fluoride (Table 2).

**Table 2.** Oral health behavioural characteristics associated with dental anxiety among medical and dental students in Arkhangelsk, Russia.

	Medical students (*n* = 422)			Dental students (*n* = 285)		
	*n*	DAS score (SD)	p*	*n*	DAS score (SD)	p*
**Regularity of dental visits**			0.040			<0.001
Irregular	116	9.39 (3.43)		43	8.12 (2.91)	
Regular	306	8.59 (3.12)		242	6.48 (2.16)	
**Tooth-brushing**			0.112			0.061
<Twice a day	104	9.11 (2.89)		38	7.50 (2.74)	
≥Twice a day	318	8.71 (3.33)		247	6.61 (2.28)	
**Skipping tooth-brushing**			0.989			0.294
No	262	8.80 (3.24)		205	6.78 (2.27)	
Yes	160	8.83 (3.22)		80	6.60 (2.58)	
**Toothpaste**			0.659			0.314
Without fluoride/difficult to answer	252	8.77 (3.24)		124	6.81 (2.21)	
With fluoride	170	8.87 (3.21)		161	6.66 (2.47)	

DAS: Dental Anxiety Scale; SD: Standard Deviation. *p from the Mann-Whitney U test.

Compared to dental students, medical students more often reported poor oral health, experienced pain in their mouths and experienced gum bleeding during tooth-brushing (45.3% vs. 25.6%, *p* < 0.001; 53.3% vs. 34.0%, *p* < 0.001; 47.9% vs. 36.5%, *p* = 0.003, respectively). Medical students who reported poor general health had a higher DAS score compared to those who reported good general health, while there were no differences in dental students. No statistically significant differences in DAS score were observed between categories of self-assessed psychological health, coping with different aspects of life, and experiencing gum bleeding during tooth-brushing. Both medical and dental students who reported poor oral health or who had experienced pain in their mouths had higher DAS scores (Table 3).

**Table 3.** Self-assessed general and oral health characteristics associated with dental anxiety among medical and dental students in Arkhangelsk, Russia.

	Medical students (*n* = 422)			Dental students (*n* = 285)		
	*n*	DAS score (SD)	p*	*n*	DAS score (SD)	p*
**Self-assessed general health**			0.016			0.150
Good	348	8.61 (3.11)		235	6.59 (2.20)	
Poor	74	9.73 (3.59)		50	7.38 (2.94)	
**Self-assessed psychological health**			0.986			0.381
Good	366	8.81 (3.24)		244	6.79 (2.40)	
Poor	56	8.79 (3.16)		41	6.37 (2.10)	
**Coping with different aspects of life**			0.213			0.670
Good	367	8.74 (3.22)		236	6.69 (2.32)	
Poor	55	9.29 (3.24)		49	6.92 (2.57)	
**Self-assessed oral health**			<0.001			0.032
Good	231	8.14 (2.81)		212	6.51 (2.18)	
Poor	191	9.62 (3.51)		73	7.34 (2.75)	
**Experienced pain in mouth**			0.015			0.014
No	197	8.35 (2.94)		188	6.40 (2.04)	
Yes	225	9.21 (3.41)		97	7.35 (2.78)	
**Experienced gum bleeding during tooth-brushing**			0.089			0.192
No	220	8.59 (3.26)		181	6.57 (2.25)	
Yes	202	9.04 (3.18)		104	6.99 (2.53)	

DAS: Dental Anxiety Scale; SD: Standard Deviation. *p from the Mann-Whitney U test.

The mean DMFT index was 7.78 (SD 4.54) and 7.31 (SD 4.34) in medical and dental students, respectively. Dental students had less DT compared to medical students (0.49 vs. 0.68; *p* = 0.020), but no differences were found in the number of MT, FT or the DMFT index. FT constituted the main fraction of dental caries experience in both medical (89.6%) and dental (91.7%) students. The OHI-S and GI were higher in medical than in dental students, (1.21 (SD 0.53) vs. 1.01 (SD 0.49), *p* < 0.001 and 0.32 (SD 0.25) vs. 0.22 (SD 0.22), *p* < 0.001, respectively). In the univariable Poisson regression, the number of MT in both groups of students, the number of DT in dental students and GI in medical students were positively associated with DAS score. For instance, every one-unit increase in MT led to a 16% increase in DAS score in dental students. No differences in DAS score by number of FT, DMFT index or OHI-S were found in medical or in dental students (Table 4).

**Table 4.** Clinical oral health status in association with dental anxiety among medical and dental students in Arkhangelsk, Russia.

	Medical students (*n* = 422)		Dental students (*n* = 285)	
	Crude IRR (95% CI)	p*	Crude IRR (95% CI)	p*
DT	1.03 (1.00–1.06)	0.059	1.06 (1.01–1.11)	0.030
MT	1.10 (1.02–1.18)	0.013	1.16 (1.06–1.27)	0.001
FT	1.00 (0.99–1.01)	0.477	1.00 (0.99–1.01)	0.880
DMFT	1.01 (1.00–1.01)	0.104	1.01 (0.99–1.02)	0.319
OHI-S	1.05 (0.98–1.11)	0.152	0.97 (0.88–1.06)	0.463
GI	1.15 (1.01–1.31)	0.029	0.88 (0.71–1.08)	0.220

IRR: incidence rate ratio; CI: confidence interval; DAS: Dental Anxiety Scale; DT: Decayed Teeth; MT: Missing Teeth due to caries; FT: Filled Teeth; DMFT: Decayed Missing and Filled Permanent Teeth; OHI-S: Simplified Oral Hygiene Index; GI: Gingival Index; SD: Standard Deviation. *p from simple Poisson regression (DAS score is the dependent variable).

The variables which remained in the multivariable Poisson analysis with DAS score as the dependent variable showed that a poor self-assessed oral health, lower mother’s education and sex (females) were associated with higher DAS score in medical students. For instance, medical students who reported poor oral health had an adjusted DAS score that was 1.15 (95% CI: 1.08–1.23) times higher than that found in those with good self-assessed oral health. In dental students, being female, reporting irregular dental visits and infrequent tooth-brushing, having experienced pain in one’s mouth or having a higher number of MT due to caries were independently associated with a higher mean DAS score. All variables in the final models explained 12.7% of the variation in the response variable in both medical and dental students (Table 5).

**Table 5.** Association between DAS score and independent variables in multivariable Poisson regression among medical and dental students in Arkhangelsk, Russia.

Variables	Medical students (*n* = 422)		Dental students (*n* = 285)	
	Adjusted IRR (95% CI)	p*	Adjusted IRR (95% CI)	p**
**Sex**		0.013		0.001
Male	Reference		Reference	
Female	1.11 (1.02–1.20)		1.16 (1.06–1.26)	
**Mother’s education**		0.001		
University	Reference			
<University	1.13 (1.05–1.20)			
**Regularity of dental visits**		0.057		0.001
Regular	Reference		Reference	
Irregular	1.07 (1.00–1.15)		1.19 (1.07–1.32)	
**Tooth-brushing**				0.007
≥Twice a day			Reference	
<Twice a day			1.17 (1.04–1.32)	
**Self-assessed general health**				0.176
Good			Reference	
Poor			1.07 (0.97–1.19)	
**Self-assessed oral health**		<0.001		
Good	Reference			
Poor	1.15 (1.08–1.23)			
**Experienced pain in mouth**		0.163		0.031
No	Reference		Reference	
Yes	1.05 (0.98–1.12)		1.09 (1.01–1.18)	
**DT**			1.03 (0.99–1.08)	0.119
**MT**			1.13 (1.03–1.24)	0.007
**GI**	1.11 (0.97–1.27)	0.121		

DAS: Dental Anxiety Scale; IRR: incidence rate ratio; CI: confidence interval; DT: Decayed Teeth; MT: Missing Teeth due to caries; GI: gingival index. *p from the final multivariable Poisson regression with backward stepwise selection of variables; Cragg & Uhler’s *R* square = 12.7%; Experienced gum bleeding during tooth-brushing, Simplified Oral Hygiene Index, DMFT index, Place of childhood residence, Tooth-brushing, Self-assessed general health, Missing teeth due to caries, Decayed teeth, Subjective socioeconomic status were removed from the final model; **p from the final multivariable Poisson regression with backward stepwise selection of variables; Cragg & Uhler’s *R* square = 12.7%; Experienced gum bleeding during tooth-brushing, Self-assessed oral health, Eligible for free education were removed from the final model.

## Discussion

The present study found that both the prevalence of high DA and mean DAS score were higher in medical than in dental students of the NSMU. In medical students, DAS score was positively associated with sex (females), lower mother’s education and poor self-assessed oral health. In dental students, sex, irregular dental visits, infrequent tooth-brushing, experienced pain in mouth and a higher number of MT due to caries were found to be significant factors associated with higher DA.

Researchers have used global questions [19], different scales [15,16,24,26] or different DAS score cut-offs to assess DA [21], which may complicate the comparability of these studies with our results. The DAS and MDAS are the most frequently used tools to measure DA in university students. The MDAS includes one additional question about anxiety of dental injection, while the other four questions are identical to those in the DAS. This item on injection will probably also reflect general syringe phobia among respondents and blend in with the total score. As the distribution of any kind of phobia is unknown in the young population of North-West Russia, we considered the DAS to be the most appropriate measurement for the present population of medical and dental students. In addition, conversion tables can be used to compare our findings with results of MDAS from other studies [36]. Nonetheless, levels of DA in our medical and dental students were found to be lower [16,18,21,23] or comparable [15] with that reported in studies among other medical or dental students.

In the present study, the dental students had a significantly lower level of DA compared to the medical students. This was expected and is in agreement with results from other studies [16,17,21]. One obvious explanation is that the level of knowledge about dentistry, severity of dental diseases and possible inconvenience while receiving dental treatment is higher among dental students. They get more information about DA during their training, they learn how to communicate with fearful dental patients and help them cope with DA, which may result in a better understanding of their own DA as well as help them cope with it. Our findings may also indicate that the curriculum of medical studies at the NSMU does not include enough information on dental diseases and treatment.

Female students from both faculties showed higher DAS scores than men, which is in line with previous studies [16,19–22]. It has been postulated that women are more susceptible to perceived threats or danger, and that they may describe their fears more openly; while men may be more emotionally stoic and hide their anxieties [37]. Nevertheless, some studies found no sex differences [15,23,24] and mentioned cultural characteristics as a possible explanation [24].

In medical students, mother’s education was associated with DA both in univariable and multivariable analysis: students whose mothers had a lower level of education had higher DAS scores. We did not find any comparable results from other studies of medical and dental students; a similar association between education and DA was found among adults in Iceland [12], while differences in DA according to parents’ education level were not statistically significant in 18-year-old Norwegian students [38]. More educated parents, characterised by high SES and less oral health problems, may maintain a positive attitude towards dentists and dental treatment indirectly, through their own experience [7]. Moreover, in medical students a higher SES was associated with a lower DA, although the association was statistically significant only in the univariable analysis. In contrast, in dental students, whose mothers were more educated compared to medical students, we did not observe any differences in DA according to level of mother’s education. It also cannot be ruled out that dental students base their attitudes on their own knowledge and experience, and less on any transferred skepticism.

Poor self-assessed oral health status was significantly associated with higher DAS scores in medical students in both univariable and multivariable analysis. In dental students, we also found a similar association in univariable analysis, although after adjustment for other factors these differences were no longer significant. Poor self-assessed oral health may reflect dental problems students may have, which in turn may result in DA. This corresponds to findings from other studies [8,9].

Our study has shown that irregular dental visits is a significant predictor of higher DA, which is in line with previous studies [22,25]. In addition, less frequent tooth-brushing was associated with a higher DAS score, which was also reported in previous studies of undergraduate students [19]. In agreement with prior studies [10], our study did not support the hypothesis that students who avoid dental visits develop good oral health habits on their own. Nevertheless, we did not find any differences in DA based on high-risk behaviours like skipping tooth-brushing or using toothpaste without fluoride. Interestingly, dental visits and frequency of tooth-brushing remained as statistically significant in the final multivariable model for dental students only. We did not find obvious explanations for these results, although one may speculate that proximity to scientific knowledge on good dental health, and resultant differences in oral health behaviour between medical and dental students, might partly explain these findings.

When data from clinical dental examinations were considered, a higher number of DT in dental students and MT in medical and dental students were associated with a higher DAS score, but after adjustment, only MT remained as a significant predictor of DAS score in the multivariable model in dental students. Moreover, having experienced pain in the mouth was an independent significant factor of a higher DA in dental students. The model of the vicious cycle of dental fear, postulated by Armfield et al. in 2007, hypothesised that “people with high dental fear are more likely to delay treatment, leading to more extensive dental problems and symptomatic visiting patterns which feed back into the maintenance or exacerbation of existing dental fear” [9]. Although causality in the present study cannot be established, and we only assessed factors associated with DA, one might assume that our findings are in line with this model. We cannot exclude the possibility that poor oral health habits in combination with irregular dental visits may have led to toothache and subsequent, painful tooth extractions. On the other hand, our sample is first and foremost characterised by high FT values, but we did not find any differences in the DAS score by the number of FT in medical or dental students. Nevertheless, DA in our study showed a better association with components of DMFT (in our case, MT due to caries) than with gingivitis. GI in medical students was significantly associated with DA in univariable analysis, but became an insignificant variable after adjustment. Gum inflammation in young adults is usually accompanied by gum bleeding only and is unlikely to result in pain. In contrast, extraction of teeth due to caries is more likely to be associated with inflammation and pain than gum problems or even restorative treatment (F component) that may lead to DA.

Given the relatively low prevalence of DA and high frequency of regular dental visits observed in the present study of medical and dental students at the NSMU, one might speculate that DA is not an obvious explanation of poor oral health in this population [29]. Nevertheless, our findings regarding factors associated with DA agree with those of other international studies. Taking into account the substantially lower level of DA in dental students than in medical students and the factors associated with DA in the two student groups investigated, public health measures should be focus on promoting dental literacy, increasing knowledge on the prevention of dental diseases, and motivating good oral health habits in young adults in North-West Russia.

### Strengths of the study

This is the first study in North-West Russia to investigate DA and factors associated with DA in young adults aged 18–25 years. We applied Corah’s DAS, an instrument commonly used for adults [6,7], and the results provide evidence of face and criterion validity for the DAS questions. Good internal consistency for the DAS was also determined. Oral health status was assessed clinically and reliability tests showed good consistency of the obtained clinical data.

### Limitations of the study

This is a cross-sectional study; thus, no causal relationships in the association between DA and the factors studied or trends in the prevalence of DA over time can be determined. Our study may be limited by the fact that only university medical and dental students of the NSMU participated in the study, which makes it challenging to generalise our findings to the young Russian population at large in North-West Russia. Moreover, our sample was not balanced with respect to response from the two student groups investigated, with a lower response rate in medical students (57.6%) compared to dental students (79.3%) in Stage 2. This may have led to an underestimation of DA and oral health problems in medical students. Although the DAS seems to have acceptable psychometric properties in the Russian version, a more thorough testing of the instrument’s reliability and validity is warranted. Nevertheless, some researchers maintain that Corah’s DAS does not consider the theoretical structure of DA and that its response categories are not mutually exclusive [6]. Only visual and tactile methods were applied during the dental examination, no radiographs were taken, which could lead to an underestimation of dental caries. Information on oral health behaviours, SES, general health and psychological health in the present study was self-reported; thus, the possibility of social desirability bias due to under- or over-reporting cannot be ruled out.

## Conclusions

In general, medical and dental students at the NSMU have a lower prevalence of high DA and lower DAS scores, as measured with the translated Russian version of Corah’s DAS, than most other medical and dental students. Level of DA was higher in medical than in dental students. DAS score in medical students was positively associated with sex (females), lower mother’s education and poor self-assessed oral health. In dental students, being female, irregular dental visits, infrequent tooth-brushing, experienced pain in the mouth and a higher number of MT were found to be significant, independent factors associated with higher DA. Public health measures should be focus on promoting dental literacy, increasing knowledge on the prevention of dental diseases and motivating good oral health habits in young adults in North-West Russia.
